# Meta-analysis of the association between toll-like receptor gene polymorphisms and hepatitis C virus infection

**DOI:** 10.3389/fmicb.2023.1254805

**Published:** 2023-10-05

**Authors:** Yuxuan Du, Shumin Li, Xinyu Wang, Jialu Liu, Yan Gao, Weimiao Lv, Ping Liu, Haiyan Huang, Junwen Luan, Leiliang Zhang

**Affiliations:** ^1^School of Clinical and Basic Medical Sciences & Institute of Basic Medical Sciences, Shandong First Medical University & Shandong Academy of Medical Sciences, Jinan, China; ^2^School of Public Health, Shandong First Medical University & Shandong Academy of Medical Sciences, Jinan, China

**Keywords:** hepatitis C (HCV), virus, toll-like receptor (TLR), single nucleotide polymorphisms (SNP), meta-analysis

## Abstract

**Objective:**

The objective of this study is to investigate the association between toll-like receptor (TLR) 3/7 gene polymorphisms and the infection by hepatitis C virus (HCV).

**Methods:**

PubMed, Embase, Web of Science, Scopus, CNKI, Wanfang Data, and SinoMed were searched to identify studies focusing on the association between the TLR3 rs3775290 or the TLR7 rs179008 single nucleotide polymorphisms (SNPs) and the HCV infection. All the related articles were collected from the inception of each database to 15 January 2023. Our meta-analysis was conducted using the allelic model, the dominant model, and the recessive model. Outcomes were presented by odds ratio (ORs) and 95% confidence interval (95%CI). The heterogeneity across studies was assessed by the I^2^ test. A subgroup analysis was performed to explore the source of heterogeneity. Funnel plots were drawn to assess the risk of publication bias. Review Manager 5.4 was used for statistical analysis.

**Results:**

Ten articles were finally included, among which six studies were analyzed for rs3775290 and five studies were analyzed for rs179008. Studies relating to rs3775290 included 801 patients and 1,045 controls, whereas studies relating to rs179008 included 924 patients and 784 controls. The results of the meta-analysis showed that there is no significant association between rs3775290 gene polymorphism and HCV infection (T vs. C: OR = 1.12, 95%CI 0.97–1.30; TT+CT vs. CC: OR = 1.20, 95%CI 0.73–1.96; TT vs. CT+CC: OR = 1.13, 95%CI 0.68–1.89). The recessive model showed that rs179008-T allele homozygotes had an 89% increased risk of infection by HCV compared with rs179008-A allele carriers (TT vs. AT+AA: OR = 1.89, 95%CI 1.13–3.16). The results of the subgroup analysis demonstrated that the characteristics of the control population may serve as an important source of heterogeneity. In the African populations, individuals with homozygous rs179008-T alleles had a higher risk of infection by HCV than rs179008-A allele carriers (OR = 2.14, 95%CI 1.18–3.87). We did not find that this difference existed in the European populations (OR = 1.24, 95%CI 0.43–3.56).

**Conclusion:**

There is no significant association between rs3775290 single nucleotide polymorphism and the infection by HCV. Individuals with homozygous rs179008-T alleles have a higher risk of an infection by HCV than rs179008-A allele carriers, which is statistically significant in the African populations.

## Introduction

Hepatitis C is a major infectious disease caused by persistent infections of human hepatocytes by hepatitis C virus (HCV), which can lead to liver fibrosis, cirrhosis, and liver cancer. Dramatic breakthroughs have been made in the use of direct-acting antiviral agents (DAAs) for the treatment of hepatitis C. However, more than 58 million people worldwide are still infected with HCV. Among patients with the three types of viral hepatitis (hepatitis A, B, and C), HCV-infected patients accounted for the greatest number of deaths and the highest mortality rate (Basit et al., 2023). HCV is widely spread worldwide, but no vaccine has emerged so far to prevent hepatitis C. Therefore, assessing the difference in the rates of HCV infections among different populations is of vital significance for the precise treatment of patients suffering from HCV-induced hepatitis or liver cancer, both theoretically and practically.

Toll-like receptors (TLRs) belong to the innate immune receptor family and play an important role in activating innate immunity, regulating the expression of cytokines, activating the adaptive immune system indirectly, and recognizing pathogen-associated molecular patterns (Hedayat et al., 2011). There are 10 family members of TLRs that exist in humans. Some of these TLRs are located on the cell membrane, while others are located on intracellular endosomes, such as TLR3, TLR7, TLR8, and TLR9. TLRs are expressed in different immune cells, such as dendritic cells (DCs), macrophages, natural killer (NK) cells, and adaptive immune cells (T and B cells) (Lester and Li, 2014). It has been demonstrated that different TLRs recognize different pathogen-associated molecular patterns, with TLR3 predominantly recognizing dsRNA (Alexopoulou et al., 2001), whereas TLR7 predominantly recognizes ssRNA (Kawai and Akira, 2011). As one of the best-studied pathogen recognition receptor families, the toll-like receptor family plays a key role in infections by HCV (Kayesh et al., 2022).

To date, there are a huge number of clinical studies on the association between single nucleotide polymorphisms (SNPs) of TLR3/7 genes and HCV-induced liver diseases, of which studies on TLR3 rs3775290 SNP and TLR7 rs179008 SNP occupy a large proportion. However, the conclusions obtained are not consistent. Therefore, we comprehensively evaluated related studies on the association between TLR3/7 gene SNPs and infections by HCV through meta-analysis to reveal the differences in the susceptibility to HCV of different individuals, providing clues for precise treatment and prognostic evaluation of HCV-infected individuals.

## Materials and methods

### Search strategy

**Figure 1:**
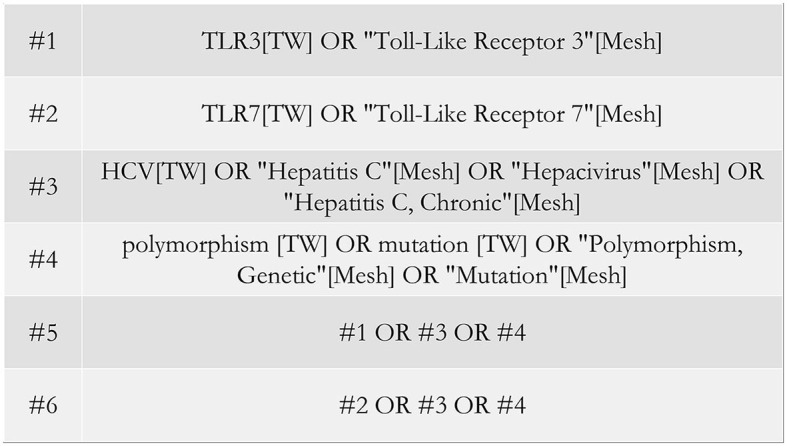
Search formulas in PubMed.

We searched PubMed, Embase, Web of Science, Scopus, CNKI, Wanfang Data, and SinoMed to collect associated studies on TLR gene polymorphisms and infections by HCV, with the last search term on 15 January 2023. Search terms included “TLR,” “Toll-Like Receptor,” “HCV,” “Hepatitis C,” “Hepacivirus,” “polymorphism,” and “mutation.” Taking PubMed as an example, the specific search formulas are shown in Figure 1.

### Inclusion and exclusion criteria

Inclusion criteria are as follows: (1) published articles whose full text is accessible; (2) case–control study or cohort study involving the relationship between TLR3 rs3775290 or TLR7 rs179008 single nucleotide polymorphisms (SNPs) and infections by HCV; (3) articles that we can extract the frequency of related genes through the original literature, authors, or relevant departments; (4) articles that we can obtain required OR value and 95%CI from the original data or by mathematical conversions.

Exclusion criteria are as follows: (1) articles that are not related to TLR3, TLR7, SNPs, or HCV-infected clinical populations; (2) studies of animal experiments; (3) articles that are unable to access the full text; (4) repeated publications; (5) articles without complete data, for instance, allele or genotype information is missing or unable to calculate.

### Data extraction and quality assessment

Two investigators searched each database with the same search strategy and screened the articles according to the inclusion and exclusion criteria independently. Then, they extracted data and assessed the quality of the included articles. Data items are as follows: (1) first author; (2) publication year; (3) country of the study population; (4) SNP information (allele and genotype frequencies) and sample sizes of the HCV group and control group; (5) type of study design; (6) source of control; (7) genetic testing method. Quality assessment of each study was performed using the Newcastle–Ottawa Scale (NOS) (Stang, 2010). The NOS consists of three parts: selection of study subjects, comparability between the groups, and measurement of exposure factors; the full scores of each part are 4, 2, and 3 points, respectively. Based on the highest score of 9 points, studies with scores of 7 points and above are considered as high-quality studies. Disagreements between the two investigators during the process of data extraction and quality assessment were resolved by a discussion with the third party.

### Statistical analysis

Hardy–Weinberg equilibrium (HWE) testing was conducted on the control group of each study using the χ2 test. Applying the allelic model, the dominant model, and the recessive model, our meta-analysis was performed, and the outcomes were presented by odds ratio (OR) and 95% confidence interval (95% CI). Heterogeneity across each included study was evaluated using the I^2^ statistic (Higgins and Thompson, 2002). A fixed-effect model was used when the heterogeneity among studies was not significant (I^2^ < 50%), otherwise a random-effect model was used (I^2^ > 50%), following a subgroup analysis to explore the source of heterogeneity. Funnel plots were drawn to assess the publication bias. All statistical analyses above were completed by Review Manager 5.4 except for HWE tests.

## Results

### Search results

**Figure 2:**
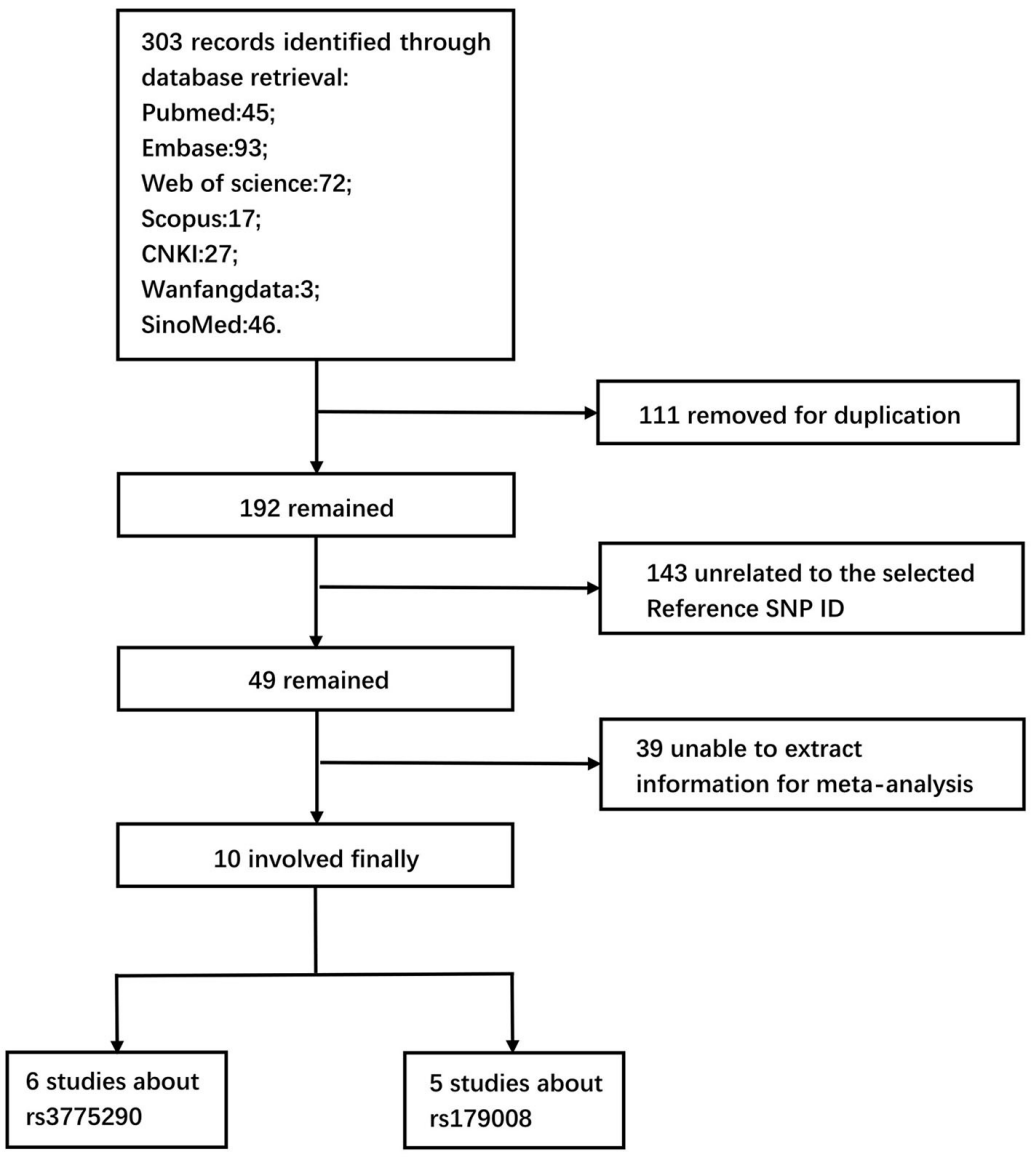
Flow diagram of retrieval and screening processes.

Seven databases, namely, PubMed, Embase, Web of Science, Scopus, CNKI, Wanfang Data, and SinoMed were initially retrieved to obtain 303 potentially relevant articles. Ten articles (Sizova et al., 2016; Chi et al., 2017; Fakhir et al., 2017; Valverde-Villegas et al., 2017; Zayed et al., 2017; Hamdy et al., 2018; Malov et al., 2018; Mosaad et al., 2018; Sghaier et al., 2018; Abdelwahab et al., 2020) were involved in the meta-analysis, including six studies about rs3775290 and five studies about rs179008, after removing 111 duplicate articles, 143 articles unrelated to the selected Reference SNP ID (rs), and 39 articles that could not extract information for the meta-analysis. The literature retrieval and screening process are presented in Figure 2.

### Study characteristics and quality assessment results

The basic characteristics of studies involved in the meta-analysis are shown in Table 1, including allele and genotype distributions. The selected studies contain populations from seven countries, covering many regions, such as Africa, Europe, and Asia. All of them are case–control studies. Two populations do not meet the standards of HWE balance (Valverde-Villegas et al., 2017; Malov et al., 2018). The quality assessment results of involved studies using the Newcastle–Ottawa Scale are presented in Table 2.

**Table 1:** Characteristics of studies involved in the meta-analysis.

**Study**	**Country**	**Controls**	Sample size^*^	**Distribution of genotype and allele**					**Genotyping method**	**P_HWE_**
**rs3775290(TLR3)**				**CC**	**CT**	**TT**	**C**	**T**		
Mosaad et al. (2018)	Egypt	Healthy individuals	100/100	6/37	90/50	4/13	102/124	98/76	PCR-RFLP	0.541
Abdelwahab et al. (2020)	Egypt	Seronegative health-care workers	70/159	46/105	18/46	6/8	110/256	30/62	PCR-RFLP	0.323
Sghaier et al. (2018)	Tunisia	Seronegative individuals	174/360	77/157	51/149	46/54	205/463	143/257	PCR-RFLP	0.062
Hamdy et al. (2018)	Egypt	Seronegative health-care workers	235/284	141/170	78/95	16/19	360/435	110/133	PCR-RFLP	0.257
Zayed et al. (2017)	Egypt	Healthy individuals	100/100	66/55	28/39	6/6	160/149	40/51	PCR-RFLP	0.791
Chi et al. (2017)	China	Healthy individuals	122/42	37/12	41/16	44/14	115/40	129/44	SNPscanTM Multiplex SNP Typing Kit	0.126
**rs179008(TLR7)**				**AA**	**AT**	**TT**	**A**	**T**		
Mosaad et al. (2018)	Egypt	Healthy individuals	30/30	13/11	10/14	7/5	144/138	56/62	PCR-RFLP	0.880
Fakhir et al. (2017)	Morocco	Individuals without liver diseases	246/72	85/33	83/27	78/12	312/133	346/77	TaqMan allelic discrimination assays, PCR-RFLP	0.123
Valverde-Villegas et al. (2017)	Brazil (European descendants)	HIV-infected individuals	16/81	10/57		6/24	22/64	2/13	PCR-RFLP	0.802
	Brazil (African descendants)	HIV-infected individuals	19/45	12/33		7/12	32/43	6/13	PCR-RFLP	0.050
Malov et al. (2018)	Russia	Healthy individuals	120/132	99/119	15/10	6/3	213/248	27/16	AmpliSense-HCV-genotype kit, real-time PCR	0.000
Sizova et al. (2016)	Ukraine	Healthy individuals	125/85	102/63	21/19	2/3	225/145	25/25	PCR-RFLP	0.460

* HCV group/control group; PCR-RFLP, PCR-restriction fragment length polymorphism; some P_*HWE*_ values were obtained directly from the original text of the study, the investigator calculated it and retained three decimal places when there was no information of P_*HWE*_ value in the original text, P_*HWE*_ < 0.05 indicates that it does not conform to Hardy-Weinberg equilibrium; The TLR7 rs179008 gene polymorphism occurs on the X chromosome, so only the female population is used as study objects in principle when counting the genotype distribution; In Valverde-Villegas et al. (2017) studies, the sample size of genotype AT and TT only showed the sum of these two because the data provided in the original article was not detailed.

**Table 2:** Quality assessment of involved studies.

**References**	**Selection of study subjects**	**Comparability between groups**	**Measurement of exposure factors**	**NOS score**
**rs3775290(TLR3)**				
Mosaad et al. (2018)	4	1	2	7
Abdelwahab et al. (2020)	4	1	2	7
Sghaier et al. (2018)	4	1	2	7
Hamdy et al. (2018)	4	1	2	7
Zayed et al. (2017)	4	1	3	8
Chi et al. (2017)	4	1	3	8
**rs179008(TLR7)**				
Mosaad et al. (2018)	4	2	3	9
Fakhir et al. (2017)	4	2	3	9
Valverde-Villegas et al. (2017)	4	1.5	3	8.5
Malov et al. (2018)	4	1	3	8
Sizova et al. (2016)	3.5	1	3	7.5

Literature quality assessment was completed independently by two investigators, and the scores shown in the table are the mean values.

Rs3775290 of TLR3 is located on the human autosome. Six studies on rs3775290 contain 1,846 subjects, including 801 patients and 1,045 controls. Rs179008 of TLR7 is located on the human X chromosome. In principle, the allelic model is based on all the subjects involved, while the dominant model and the recessive model are based only on female subjects. The study by Valverde-Villegas et al. (2017) is an exception for the allelic model, only focused on males of the subjects according to the original data. Five studies on rs179008 contain 1,001 female subjects including 556 patients and 445 controls and 707 male subjects including 368 patients and 339 controls.

### Meta-analysis results

**Figure 3:**
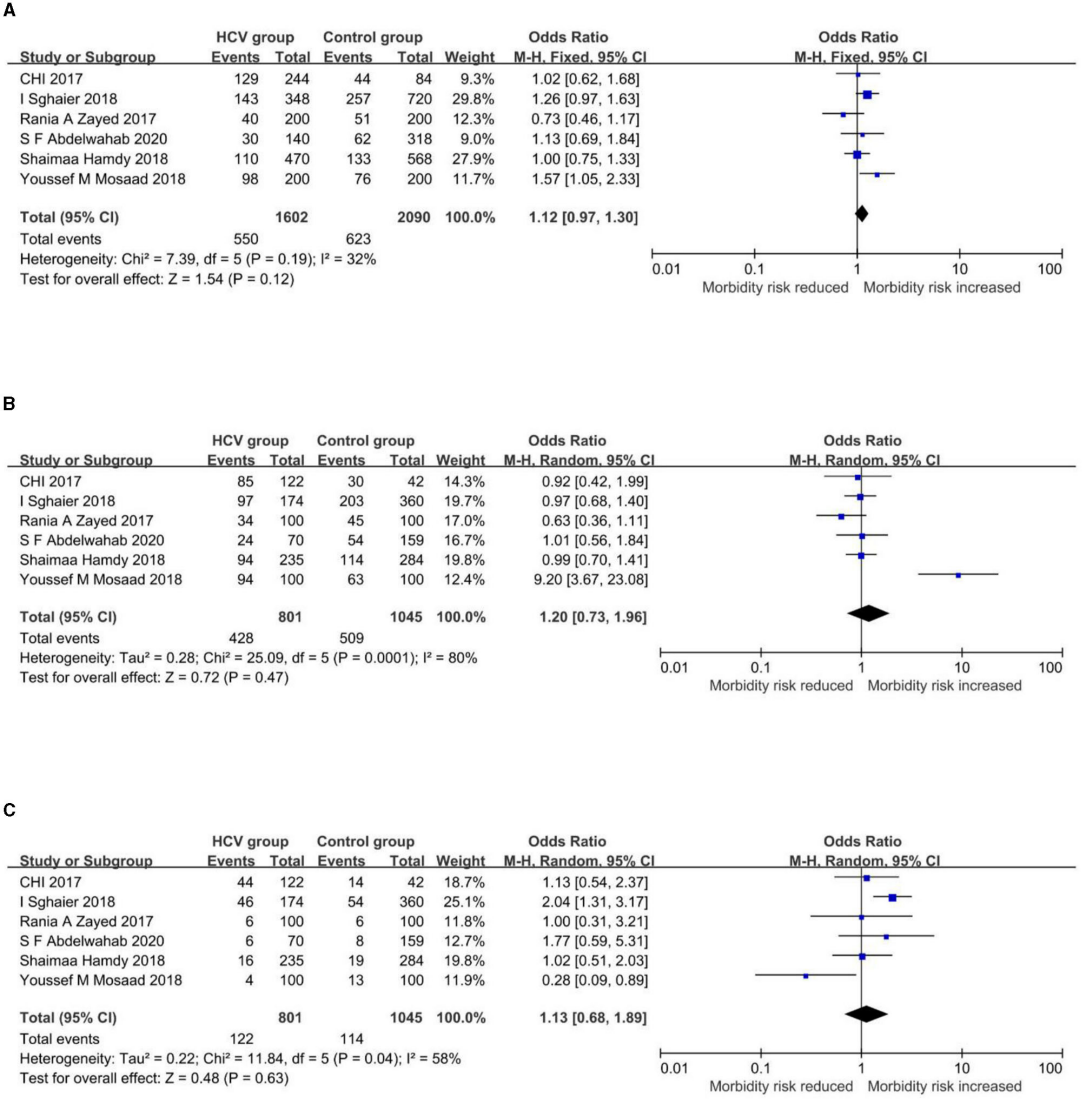
Forest plots of the association between rs3775290 and HCV infection. **(A)** Allelic model (T vs. C). **(B)** Dominant model (TT+CT vs. CC). **(C)** Recessive model (TT vs. CT+CC).

**Figure 4:**
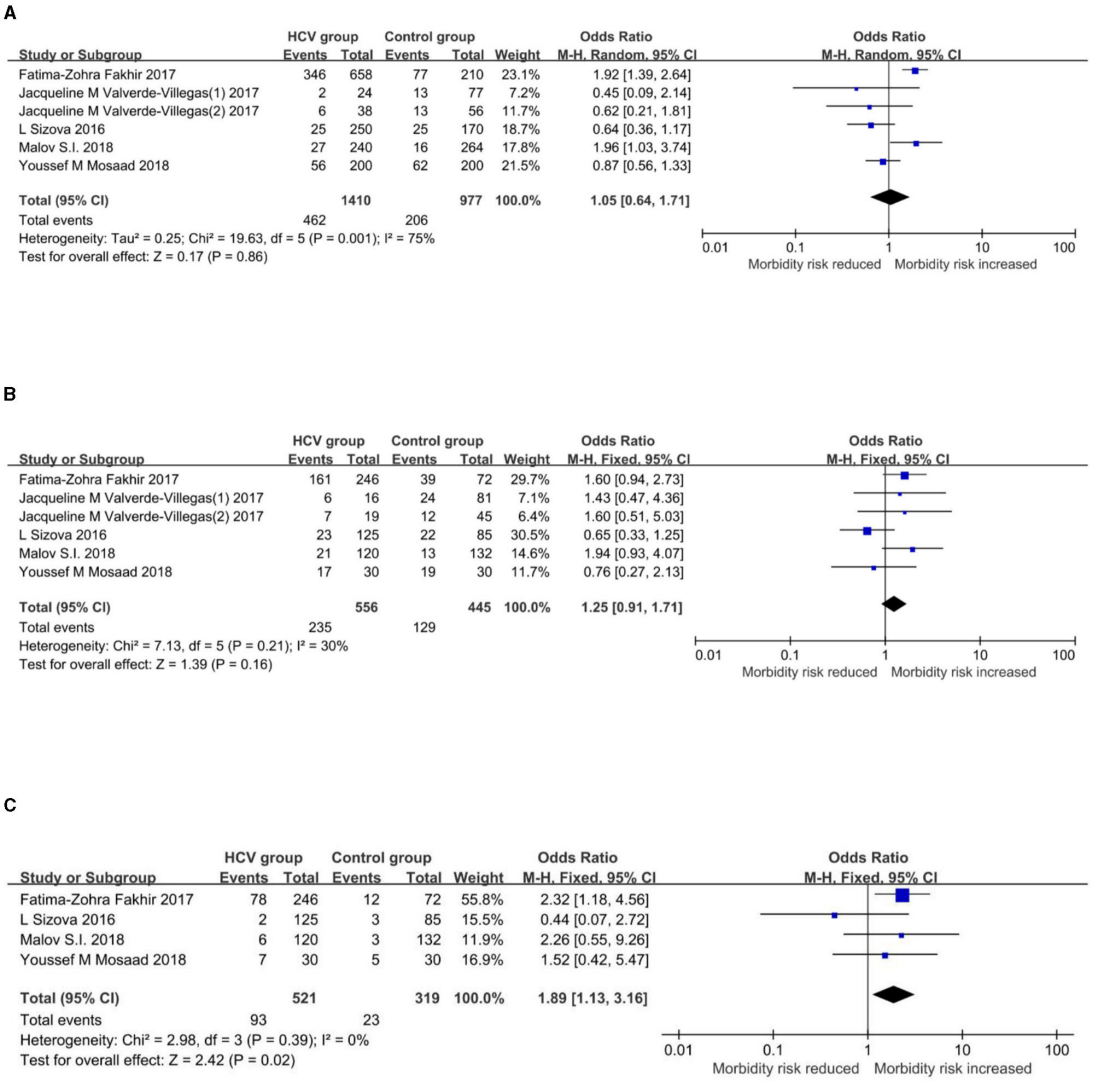
Forest plots of the association between rs179008 and HCV infection. **(A)** Allelic model (T vs. A). **(B)** Dominant model (TT+AT vs. AA). **(C)** Recessive model (TT vs. AT+AA).

Meta-analysis results of the association between TLR3/7 gene polymorphisms and infections by HCV are summarized in Table 3. Forest plots of each genetic model are shown in Figures 3, 4.

**Table 3:** Meta-analysis results of the association between TLR gene polymorphisms and HCV infection.

**Genetic model**	**Number of studies**	**HCV group/** **control group**	**I^2^**	**Statistical effect model**	**OR,95%CI**
**rs3775290**					
Allelic model (T vs. C)	6	1602/2090	32%	F	1.12[0.97,1.30]
Dominant model (TT+CT vs. CC)	6	801/1045	80%	R	1.20[0.73,1.96]
Recessive model (TT vs. CT+CC)	6	801/1045	58%	R	1.13[0.68,1.89]
**rs179008**					
Allelic model (T vs. A)	6	1410/977	75%	R	1.05[0.64,1.71]
Dominant model (TT+AT vs. AA)	6	556/445	30%	F	1.25[0.91,1.71]
Recessive model (TT vs. AT+AA)	4	521/319	0%	F	1.89[1.13,3.16]

I^2^, the Inconsistency statistics; OR, odds ratio; CI, confidence interval; F, fixed-effect model; R, random-effect model.

#### Forest plots of genetic models and their interpretations

Allele frequencies of rs3775290-T are 34.33% and 29.81% in the HCV group and control group, respectively. In the allelic model (Figure 3A), the fixed-effect model showed a pooled OR and 95%CI of 1.12[0.97, 1.30], with the allele T as the exposure factor and the allele C as the non-exposure factor. In the dominant model (Figure 3B), the random-effect model showed a pooled OR and 95%CI of 1.20[0.73, 1.96], with the genotype TT+CT as the exposure factor and the genotype CC as the non-exposure factor. In the recessive model (Figure 3C), the random-effect model showed a pooled OR and 95%CI of 1.13[0.68, 1.89], with the genotype TT as the exposure factor and the genotypes CT+CC as the non-exposure factor. As a consequence, the association between this *TLR3* gene polymorphism and the infection by HCV cannot yet be considered statistically significant. The details of the results above are shown in Figure 3.

Allele frequencies of rs179008-T are 32.77% and 21.08% in the HCV group and control group, respectively. In the allelic model (Figure 4A), the random-effect model showed a pooled OR and 95%CI of 1.05[0.64, 1.71], with the allele T as the exposure factor and the allele A as the non-exposure factor. In the dominant model (Figure 4B), the fixed-effect model showed a pooled OR and 95%CI of 1.25[0.91, 1.71], with the genotypes TT+AT as the exposure factor and the genotype AA as the non-exposure factor. In the recessive model (Figure 4C), the fixed-effect model showed a pooled OR and 95%CI of 1.89[1.13, 3.16], with the genotype TT as the exposure factor and the genotypes AT+AA as the non-exposure factor, which indicated that the risk of an infection by HCV is increased by 89% in TT homozygotes compared with A allele carriers. The association between this *TLR7* gene polymorphism and the infection by HCV is statistically significant. The details of the results above are shown in Figure 4.

#### Subgroup analysis and sensitivity analysis

The results of the heterogeneity tests showed significant heterogeneity in the dominant model (TT + CT vs. CC, I^2^ = 80%) and the recessive model (TT vs. CT + CC, I^2^ = 58%) of the TLR3 rs3775290 gene polymorphism and in the allelic model (T vs. A, I^2^ = 75%) of the *TLR7 rs179008* gene polymorphism. A subgroup analysis was conducted to calculate pooled ORs and their 95%CIs to explore the reasons for heterogeneity due to the high heterogeneity.

The results of the subgroup analysis (Table 4) are as follows: (1) The characteristics of the control group may be a common source of heterogeneity in the study on the association between *rs3775290* or *rs179008* gene polymorphism and infections by HCV. In the process of our research, it was found that seronegative individuals generally mean that the study subjects were selected from hospitals. They are either patients from other departments of a hospital or healthcare workers who have come in contact with patients. Therefore, they are different from the ordinary healthy population. In the recessive model associated with rs3775290, when the control group is composed of non-healthy individuals, TT homozygotes have a 66% increased risk of infection by HCV compared with C allele carriers, which is statistically significant (OR = 1.66, 95%CI 1.17–2.36). However, when the control group is composed of healthy individuals, there is no statistically significant difference in the risk of infection by HCV between TT homozygotes and C allele carriers (OR = 0.73, 95%CI 0.31–1.69). In the dominant model associated with rs179008, when the control group is composed of non-healthy individuals, T allele carriers have a 57% increased risk of infection by HCV compared with AA homozygotes, which is statistically significant (OR = 1.57, 95%CI 1.01–2.45). However, when the control group is composed of healthy individuals, there is no statistically significant difference in the risk of infection by HCV between T allele carriers and AA homozygotes (OR = 1.00, 95%CI 0.65–1.55). (2) The recessive model on rs179008 showed that there is a statistically significant difference between this *TLR7* gene polymorphism and the infection by HCV. The heterogeneity of this conclusion may come from the regional distribution of the study subjects. Compared with the European population (OR = 1.24, 95%CI 0.43–3.56), a higher pooled OR was obtained in the North African population (OR = 2.14, 95%CI 1.18–3.87). It suggested that rs179008-T allele homozygotes have a higher risk of infection by HCV than A allele carriers in the North African population, but this difference cannot be considered to exist in the European population.

**Table 4:** Subgroup analysis and sensitivity analysis of the association between TLR gene polymorphism and HCV infection.

**Subgroup**	**Genetic model**	**Number of studies**	**HCV group/ control group**	**I^2^**	**Statistical effect model**	**OR,95%CI**
**rs3775290(TLR3)**						
Control group						
Healthy individuals	T vs. C	3	644/484	67%	R	1.07[0.68,1.68]
	TT+CT vs. CC	3	322/242	92%	R	1.69[0.37,7.66]
	TT vs. CT+CC	3	322/242	52%	R	0.73[0.31,1.69]
Non-healthy individuals	T vs. C	3	958/1606	0%	F	1.13[0.94,1.36]
	TT+CT vs. CC	3	479/803	0%	F	0.99[0.78,1.25]
	TT vs. CT+CC	3	479/803	27%	F	1.66[1.17,2.36]
Area						
North Africa	T vs. C	5	1358/2006	45%	F	1.13[0.97,1.32]
	TT+CT vs. CC	5	679/1003	84%	R	1.27[0.72,2.23]
	TT vs. CT+CC	5	679/1003	65%	R	1.10[0.58,2.09]
Asia	T vs. C	1	244/84	-	-	1.02[0.62,1.68]
	TT+CT vs. CC	1	122/42	-	-	0.92[0.42,1.99]
	TT vs. CT+CC	1	122/42	-	-	1.13[0.54,2.37]
**rs179008(TLR7)**						
Control group						
Healthy individuals	T vs. A	3	690/634	70%	R	1.01[0.56,1.81]
	TT+AT vs. AA	3	275/247	60%	R	1.00[0.65,1.55]
	TT vs. AT+AA	3	275/247	0%	F	1.35[0.60,3.04]
Non-healthy individuals	T vs. A	3	720/343	70%	R	0.98[0.36,2.64]
	TT+AT vs. AA	3	281/198	0%	F	1.57[1.01,2.45]
	TT vs. AT+AA	1	246/72	-	-	2.32[1.18,4.56]
Area						
North Africa	T vs. A	3	896/466	81%	R	1.11[0.56,2.21]
	TT+AT vs. AA	3	295/147	0%	F	1.40[0.90,2.16]
	TT vs. AT+AA	2	276/102	0%	F	2.14[1.18,3.87]
Europe	T vs. A	3	514/511	73%	R	0.93[0.39,2.31]
	TT+AT vs. AA	3	261/298	60%	R	1.17[0.56,2.46]
	TT vs. AT+AA	2	245/217	48%	F	1.24[0.43,3.56]
HWE (sensitivity analysis)						
Yes	T vs. A	4	1132/657	81%	R	0.96[0.51,1.81]
	TT+AT vs. AA	4	417/268	41%	F	1.09[0.76,1.57]
	TT vs. AT+AA	3	401/187	31%	F	1.84[1.06,3.20]
No	T vs. A	2	278/320	69%	R	1.20[0.39,3.67]
	TT+AT vs. AA	2	139/177	0%	F	1.84[0.99,3.42]
	TT vs. AT+AA	1	120/132	-	-	2.26[0.55,9.26]

I^2^, the Inconsistency statistics; OR, odds ratio; CI, confidence interval; F, fixed-effect model; R, random-effect model.

The results of a sensitivity analysis (Table 4) showed that the pooled OR (OR = 1.84, 95%CI 1.06–3.20) on rs179008 in the recessive model has no essential change after excluding a study whose control group does not meet the standards of Hardy–Weinberg equilibrium. It indicated that the sensitivity of our meta-analysis results is low, and the conclusion is steady. For this reason, we can agree that the association between this *TLR7* gene polymorphism and the infection by HCV is statistically significant. Indeed, the risk of an infection by HCV is higher in TT homozygotes than in A allele carriers.

### Publication bias assessment

**Figure 5:**
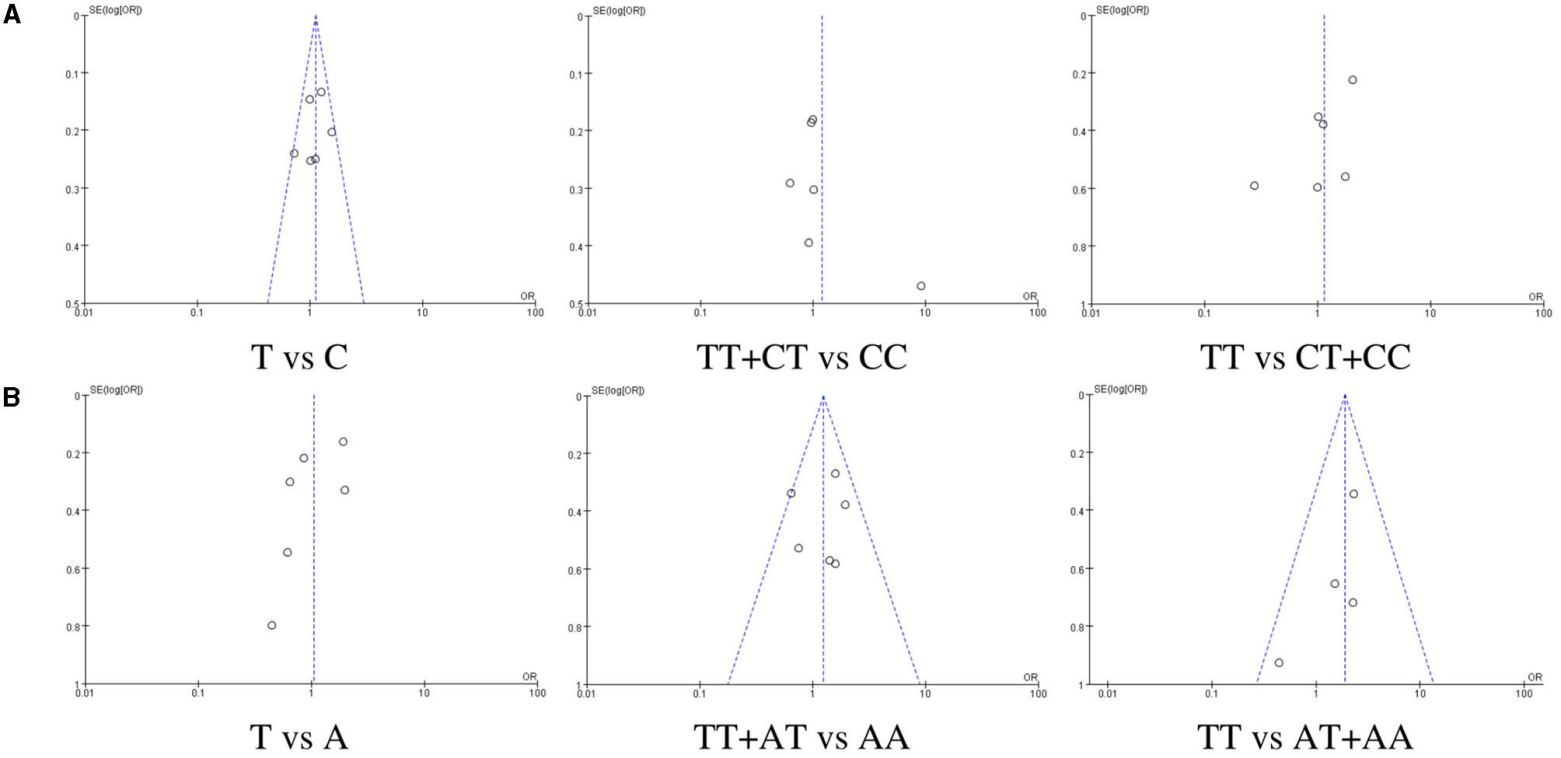
Funnel plots of publication bias. **(A)** relating to rs3775290(TLR3), **(B)** relating to rs179008(TLR7).

Publication bias was assessed by drawing funnel plots using Review Manager 5.4 software, and the results are shown in Figure 5. In studies on the TLR3 rs3775290, no significant publication bias was observed between the allelic model (T vs. C) and the recessive model (TT vs. CT+CC), but there are some publication biases in the dominant model (TT+CT vs. CC). In studies on the TLR7 rs179008, no significant publication bias was observed between the dominant model (TT+AT vs. AA) and the recessive model (TT vs. AT+AA), but there are some publication biases in the allelic model (T vs. A).

## Discussion

In this study, a meta-analysis was performed on the 10 included articles to investigate the association between toll-like receptor single nucleotide polymorphisms and infection by HCV. As the results are revealed, there is no significant association between rs3775290 of TLR3 and the infection by HCV, while the homozygous rs179008-T alleles may increase the risk of HCV infection. However, it does not mean that the rs3775290 SNP is completely unrelated to the infection by HCV. Talaat et al. (2022) suggested that rs3775290 might be associated with the development of cirrhosis in HCV-infected individuals. The research by Sghaier et al. (2017) showed a higher frequency of TLR3 rs3775290 C/C genotype in patients with sustained viral response compared with non-responders; TLR3 is involved in the pathogenesis of the infection by HCV and may be more suitable for activating antiviral programs than TLR4.

The recessive model showed that rs179008-T allele homozygotes have an 89% increased risk of an infection by HCV compared with A allele carriers. The results of the subgroup analysis demonstrated that the association between TLR7 rs179008 SNP and the infection by HCV is statistically significant in the North African population but not in the European population. However, one thing we should pay more attention to is that it is crucial for researchers to consider and address the potential genetic diversity within the study population to ensure accurate and reliable results. For instance, if a study is conducted in an African country, it is reasonable to assume that the subjects involved are predominantly of African descent. African countries typically have populations composed primarily of individuals with African ancestry. However, it is important to note that individual genetic backgrounds can vary within populations, and there may be some degree of genetic diversity even within a specific geographical region.

Due to certain commonalities of viruses, research studies on relevant viruses have guiding significance for further exploration of the mechanism of how the rs179008 SNP affects the susceptibility to HCV. Oh et al. (2009) reported that the polymorphism of Gln11Leu on TLR7, which is encoded by the rs179008-T allele, is associated with a high viral load and the progression of immunosuppression in patients with human immunodeficiency virus (HIV). Said et al. (2014) found that Gln11Leu on TLR7 may be associated with a rapid decrease in CD4T cell count during viremia caused by HIV. These two conclusions are consistent with the results of a systematic review written by Shi et al. (2020), which showed that the rs179008-T allele has a risk effect on the infection by HIV. A study by Sengupta et al. (2021) showed that the rs179008 SNP affects the prevalence and the viral load of chikungunya virus (CHIKV) and dengue virus (DENV). Compared with the wild TLR7-rs179008Q, the TLR7-rs179008L variant located in the signal peptide region is more hydrophobic and smaller, which might disturb the recognition of signal peptides. Moreover, the docking of TLR7 with SPC18 shows that the combination of the TLR7-rs179008Q variant and SPC18 is superior to the L variant, considering the change in free energy and more residues of the Q variant docking with SPC18. In future, we can focus more on the relationship between TLR7 gene polymorphism and the viral load, the immunosuppression progression and the T-cell count in patients with hepatitis C. Furthermore, an increasing number of evidence has demonstrated that B-cell lymphoproliferative disorders such as mixed cryoglobulinemia (MC) are related to HCV infection. In the research by Liao et al. (2021), they confirmed that HCV-induced GU-enriched miRNA (e.g., miR-122, let-7b, and miR-206) could upregulate the expression of B-cell activating factor (BAFF) through exosome transmission and TLR7 activation.

When the control group was composed of healthy individuals, no significant association could be considered to exist between the TLR3 rs3775290 SNP or TLR7 rs179008 SNP and the infection by HCV, based on the pooled ORs and their 95%CIs of each genetic model. However, when the selection criteria for the control group were only seronegative individuals or patients without liver disease, the pooled ORs of some genetic models showed that the rs3775290 and rs179008 are associated with the infection by HCV. The results of the subgroup analysis, according to whether the control group is composed of healthy individuals or not, suggested that there may be other potential confounding factors affecting the meta-analysis results. Mazzaro et al. (2021) found that lots of experimental and clinical data showed a causal association between extrahepatic diseases and infection by HCV, such as mixed cryoglobulinemia, non-Hodgkin lymphoma (NHL), neurological and psychiatric disorders, insulin resistance, type 2 diabetes, cardiovascular diseases, and certain rheumatic diseases. Extrahepatic diseases may influence the immune status of the body and that might be the reason why they affect the association analysis between toll-like receptor single nucleotide gene polymorphisms and the infection by HCV. In addition to hepatitis C, the rs179008 SNP is also associated with diseases such as sarcoidosis (Bordignon et al., 2013), basal cell carcinoma (BCC) (Piaserico et al., 2015; Russo et al., 2016), melanoma (Sabado et al., 2015), and asthma (Møller-Larsen et al., 2008; Törmänen et al., 2017). These diseases may have undiscovered impacts on the infection by HCV, which indirectly reflects in the association between the *TLR7* gene polymorphism and the susceptibility to HCV, interfering with our judgment of the statistical analysis results.

Pattern recognition receptors (PRRs), which can directly recognize certain specific and shared molecular structures of foreign pathogens and their products, exist on the surface of innate immune cells, on the membrane of intracellular organelles, and in the cytoplasm and blood. Toll-like receptors are members of the pattern recognition receptor family. In addition to toll-like receptors, other PRRs such as mannose receptors (Andersen et al., 2014; Gantzel et al., 2020), scavenger receptors (Grove et al., 2007; Dao Thi et al., 2011), and NOD-like receptors (Aggan et al., 2022) are also associated with HCV infection. Pathogen-associated molecular patterns (PAMPs) are ligand molecules that pattern recognition receptors recognize and bind to. Different PRRs correspond to different PAMPs, but some of their similarities suggest that these receptors may have similar effects on HCV infection, which expands new ideas for exploring the mechanism of TLRs affecting HCV infection.

Askar et al. (2010) found that the rs179008 gene polymorphism is associated with the differences in the gene expression of interleukin-29 (IL-29)/λ1 interferon (IFN-λ1) and IFN-λ receptor in the livers of HCV-infected individuals. These differences may be important for the effect of the IFN-λ-based treatment of hepatitis C. In terms of vaccines, the Rotarix vaccine is currently the most commonly used live oral rotavirus vaccine. Miya et al. (2021) believed that the TLR7 variant caused by the rs179008 SNP may affect the production of IgA antibodies and the serum conversion after receiving the Rotarix vaccine in black South African infants. It is worth noting that the above-mentioned research results can provide ideas for the transformation from our study to clinical applications.

This study has some limitations. First, the number of included studies is small. When performing the subgroup analysis related to rs3775290 of TLR3, the Asian region only referred to one article; thus, we cannot draw a reliable comprehensive conclusion. It is urgent to carry out more large-sample and high-quality studies in Asian regions, in order to continue investigating the link between the TLR3 rs3775290 gene polymorphism and infections by HCV. Second, the publication bias was evaluated based on the symmetry of funnel plots, but the visual inspection decided that the evaluation results were subjective. Apart from the publication bias, the selection bias, the language bias, and the citation bias, other factors may also affect the symmetry of the funnel plots.

In conclusion, HCV, as a single-stranded RNA virus, can be divided into six genotypes according to its different genomic nucleotide sequences. The genotype of HCV is a vital factor in predicting a sustained viral response. Except for the genotype of HCV, gene polymorphisms of the host can also affect the infection, the therapeutic effect, the prognosis of hepatitis C (Xu et al., 2013), and even the severity of a recurrence of hepatitis C after liver transplantation (Citores et al., 2016). Studies have shown that the host interleukin-28 (IL-28) (Mi et al., 2012) and the gene polymorphisms of human leukocyte antigen (HLA) (Huang et al., 2014) are associated with infections by HCV. In our study, we focused on the single nucleotide gene polymorphism of toll-like receptors, a host genetic factor. We conducted a comprehensive quantitative analysis based on the results of a number of studies to provide support for the in-depth study of the rs3775290 or the rs179008. The results enable us to have new ideas about individualized precise prevention and treatment for groups at risk of HCV.

## Author contributions

YD: Formal analysis, Writing—original draft, Writing—review and editing. SL: Data curation, Writing—original draft. XW: Data curation, Writing—original draft. JLi: Resources, Writing—original draft. YG: Resources, Writing—original draft. WL: Writing—original draft. PL: Writing—original draft. HH: Writing—review and editing. JLu: Conceptualization, Supervision, Writing—review and editing. LZ: Funding acquisition, Supervision, Writing—review and editing.
