# Corrigendum: Meta-analysis of the association between toll-like receptor gene polymorphisms and hepatitis C virus infection

**DOI:** 10.3389/fmicb.2023.1330170

**Published:** 2023-11-21

**Authors:** Yuxuan Du, Shumin Li, Xinyu Wang, Jialu Liu, Yan Gao, Weimiao Lv, Ping Liu, Haiyan Huang, Junwen Luan, Leiliang Zhang

**Affiliations:** ^1^School of Clinical and Basic Medical Sciences & Institute of Basic Medical Sciences, Shandong First Medical University & Shandong Academy of Medical Sciences, Jinan, China; ^2^School of Public Health, Shandong First Medical University & Shandong Academy of Medical Sciences, Jinan, China

**Keywords:** hepatitis C (HCV), virus, toll-like receptor (TLR), single nucleotide polymorphisms (SNP), meta-analysis

In the published article, there was an error in Table 1. We mistakenly labeled the reference by Sizova et al. (2016) as Russia. It should be corrected to Ukraine. The corrected Table 1 and its caption appear below.

**Table 1:** Characteristics of studies involved in the meta-analysis.

**Study**	**Country**	**Controls**	Sample size^*^	**Distribution of genotype and allele**					**Genotyping method**	**P_HWE_**
**rs3775290(TLR3)**				**CC**	**CT**	**TT**	**C**	**T**		
Mosaad et al. (2018)	Egypt	Healthy individuals	100/100	6/37	90/50	4/13	102/124	98/76	PCR-RFLP	0.541
Abdelwahab et al. (2020)	Egypt	Seronegative health-care workers	70/159	46/105	18/46	6/8	110/256	30/62	PCR-RFLP	0.323
Sghaier et al. (2018)	Tunisia	Seronegative individuals	174/360	77/157	51/149	46/54	205/463	143/257	PCR-RFLP	0.062
Hamdy et al. (2018)	Egypt	Seronegative health-care workers	235/284	141/170	78/95	16/19	360/435	110/133	PCR-RFLP	0.257
Zayed et al. (2017)	Egypt	Healthy individuals	100/100	66/55	28/39	6/6	160/149	40/51	PCR-RFLP	0.791
Chi et al. (2017)	China	Healthy individuals	122/42	37/12	41/16	44/14	115/40	129/44	SNPscanTM Multiplex SNP Typing Kit	0.126
**rs179008(TLR7)**				**AA**	**AT**	**TT**	**A**	**T**		
Mosaad et al. (2018)	Egypt	Healthy individuals	30/30	13/11	10/14	7/5	144/138	56/62	PCR-RFLP	0.880
Fakhir et al. (2017)	Morocco	Individuals without liver diseases	246/72	85/33	83/27	78/12	312/133	346/77	TaqMan allelic discrimination assays, PCR-RFLP	0.123
Valverde-Villegas et al. (2017)	Brazil (European descendants)	HIV-infected individuals	16/81	10/57		6/24	22/64	2/13	PCR-RFLP	0.802
	Brazil (African descendants)	HIV-infected individuals	19/45	12/33		7/12	32/43	6/13	PCR-RFLP	0.050
Malov et al. (2018)	Russia	Healthy individuals	120/132	99/119	15/10	6/3	213/248	27/16	AmpliSense-HCV-genotype kit, real-time PCR	0.000
Sizova et al. (2016)	Ukraine	Healthy individuals	125/85	102/63	21/19	2/3	225/145	25/25	PCR-RFLP	0.460

* HCV group/control group; PCR-RFLP, PCR-restriction fragment length polymorphism; some P_*HWE*_ values were obtained directly from the original text of the study, the investigator calculated it and retained three decimal places when there was no information of P_*HWE*_ value in the original text, P_*HWE*_ < 0.05 indicates that it does not conform to Hardy-Weinberg equilibrium; The TLR7 rs179008 gene polymorphism occurs on the X chromosome, so only the female population is used as study objects in principle when counting the genotype distribution; In Valverde-Villegas et al. (2017) studies, the sample size of genotype AT and TT only showed the sum of these two because the data provided in the original article was not detailed.

In the published article, there was an error. The selected studies used for our meta-analysis contain populations from seven countries, instead of six countries.

A correction has been made to Results, *Study characteristics and quality assessment results*, Paragraph 1. This sentence previously stated:

“The selected studies contain populations from six countries, covering many regions, such as Africa, Europe, and Asia.”

The corrected sentence appears below:

“The selected studies contain populations from seven countries, covering many regions, such as Africa, Europe, and Asia.”

The authors apologize for this error and state that this does not change the scientific conclusions of the article in any way. The original article has been updated.
